# On the use of multi–objective evolutionary classifiers for breast cancer detection

**DOI:** 10.1371/journal.pone.0269950

**Published:** 2022-07-19

**Authors:** Laura Dioşan, Anca Andreica, Irina Voiculescu

**Affiliations:** 1 Department of Computer Science, Babes-Bolyai University, Cluj-Napoca, Romania; 2 Department of Computer Science, University of Oxford, Oxford, United Kingdom; Universidad de Guadalajara, MEXICO

## Abstract

**Purpose:**

Breast cancer is one of the most common tumours in women, nevertheless, it is also one of the cancers that is most usually treated. As a result, early detection is critical, which can be accomplished by routine mammograms. This paper aims to describe, analyze, compare and evaluate three image descriptors involved in classifying breast cancer images from four databases.

**Approach:**

Multi–Objective Evolutionary Algorithms (MOEAs) prove themselves as being efficient methods for selection and classification problems. This paper aims to study combinations of well–known classification objectives in order to compare the results of their application in solving very specific learning problems. The experimental results undergo empirical analysis which is supported by a statistical approach. The results are illustrated on a collection of medical image databases, but with a focus on the MOEAs’ performance in terms of several well–known measures. The databases were chosen specifically to feature reliable human annotations, so as to measure the correlation between the gold standard classifications and the various MOEA classifications.

**Results:**

We have seen how different statistical tests rank one algorithm over the others in our set as being better. These findings are unsurprising, revealing that there is no single gold standard for comparing diverse techniques or evolutionary algorithms. Furthermore, building meta-classifiers and evaluating them using a single, favorable metric is both extremely unwise and unsatisfactory, as the impact is to skew the results.

**Conclusions:**

The best method to address these flaws is to select the right set of objectives and criteria. Using accuracy-related objectives, for example, is directly linked to maximizing the number of true positives. If, on the other hand, accuracy is chosen as the generic metric, the primary classification goal is shifted to increasing the positively categorized data points.

## Introduction

Breast cancer is the most frequent form of cancer and the main cause of death among women, as reported by the World Health Organization.

Even though mammograms can detect early asymptomatic stages of the disease [1], they rely greatly on the radiologist’s knowledge and can result in a high incidence of incorrect diagnoses. To limit the amount of incorrect diagnoses, a technique known as double-reading has been promoted, which involves two radiologists concurrently analyzing mammograms and then comparing the results [2]. This measure, however, increases prices and workload. Computed Aided Diagnosis (CAD) systems can help a single radiologist establish a diagnosis and provide support for his or her judgment as a solution to this challenge [3]. Machine learning classifiers are commonly used in CAD systems to infer diagnosis. A set of predictors characterizing the observations must be provided in order to train the classifier. Image descriptors (also known as visual descriptors) are numerical representations of visual features such as form, color, and texture that can be used to anticipate how images from mammograms will be described.

In this paper we present several Genetic Programming (GP) classifiers for the learning phase of a breast cancer CAD system. The input data of the classifiers will consist of characteristics extracted from images using several image descriptors, namely Statistical Moments, Haralick features and Grey Level Run Length (GLRL). The purpose of this paper is to perform an apprehensive evaluation of the performances of different GP classifiers for solving the given task, when considering multiple data sets. We decided to develop GP-based decision models because they offer a great potential for classification: the classifiers can be modeled as trees that encode complex patterns by combining various operations or functions inside that representation. Furthermore, GP, as any kind of Evolutionary algorithms, can be easily and suitably applied to multi-objective problems when several goals have to be optimized simultaneously and which are often conflicting. In this context, both single-objective and multi-objective classifiers are used, and the usage of different objectives and fitness functions is investigated. For the multi-objective scenario, two strategies for combining the classifiers are considered, namely majority voting and using GP for ensemble selection. The performance of the system is measured, depending on the classifier, either by using the general accuracy or the Wilcoxon-Mann-Whitney statistic.

Interpretability, viewed as the attribute of a classifier to be understood by a human being—a radiologist in our case—and classification accuracy, viewed as the distance between the real classifier and the modeled one, are two important features of a medical decision model. These two goals represent contradictory issues in the design of a CAD system: if one of them increases, the other one must decrease. Our GP-based models propose a solution to this Interpretability-Accuracy trade-off [4] by limiting the size of a classifier.

Moreover, this paper presents an in depth statistical analysis of the obtained results by performing Wilcoxon sign-ranks tests, Friedman tests, and Nemenyi post-hoc tests. For all of these tests, the null-hypothesis is that the considered classifiers have similar performances for the given data sets and representation models. The compared classifiers are all GP-based, with a special focus on those MO. A comparison with other non-GP classifiers (e.g. SVM or ANN) is not possible since the considered models are MO, while non-GP approaches optimise a single objective.

Image classifiers of various sorts have been developed over the years using a variety of different methods such as decision trees [5, 6], neural networks [7, 8], or support vector machines [9]. Evolutionary algorithms have gained increased popularity for classification tasks [10] due to the simplicity with which fitness functions can be expressed, and also the variety of the expressions (linear, non-linear, tree-based, etc.).

There is flexibility in the complexity of the expression which defines the classifier. At one end of the spectrum, one can focus on the readability of the ensuing classifier, writing the fitness function in such a way that a human user (e.g. a clinician) can understand the decisions made in a particular classification task. It is also possible to set a maximum depth of any derived decision tree. At the other way of the spectrum, it is also possible to pack the fitness function with many parameters, allowing the process to derive a task–adequate classifier which may well seem impenetrable.

The use of evolutionary algorithms for classification is nevertheless non–trivial in contexts which involve the interplay of several criteria [11]. For example, the user can be interested simultaneously in the runtime, the quality of the results and the accuracy of the classification. Multi–criteria problems are hard to express and solve with conventional methods but, because of the ability of fitness functions to incorporate parameters simultaneously, can be tackled effectively using evolutionary algorithms.

More often than not, real–life problems involve imbalanced data: there is no uniform gaussian distribution of any of the parameter readings. In the case of cancer diagnosis, for instance, the majority of the population is healthy, and a relatively small number of pathological cases constitute outliers in need of detection. Classification methods which rely on a uniform distribution of balanced data are ineligible in cases of data imbalance [12, 13].

In cases where a number of classification criteria are to be considered and optimised, it is impractical to design a sequential search for potential solutions: the search space can be huge, and therefore, for speed reasons, the search needs to improve simultaneously on each of the measured criteria.

## Materials and methods

We illustrate the above points using the real–life problem of breast cancer diagnosis. In particular, the reduction of the number of false positives (through automated classification) can significantly reduce physiological stress caused by unnecessary surgery [14].

An automated diagnosis system is expected to classify mammogram images into either pathological or healthy. The process starts by training the classifier on a set of annotated images representing mammographies with known (and assumed correct) diagnosis. The classification algorithm then studies these examples and learns a decision model, which it can then apply to any mammogram database not previously encountered. The main aim of the learning process is to identify patterns within observed data, get insight and build models that link the input to the output data. These models will be used to predict the output for new and unsen before inputs.

The generic problem can be formalised as described in the next paragraph. Let *I* be a set of *n* images (*I* = {***I***_**1**_, ***I***_**2**_, …, ***I***_***n***_}) represented by *m* real numbers features (***I***_***i***_ = (*I*_*i*,1_, *I*_*i*,2_, …, *I*_*i*,*m*_), for *i* = 1, 2, …, *n*). Each image is associated with a binary output label *class*_*i*_ (-1 for healthy sample and 1 for presence of pathology): *CLASS* = {*class*_1_, *class*_2_, …, *class*_*n*_}, where each *class*_*i*_, with *i* = 1, 2, …, *n* belongs to {−1, 1}. The number of images from the positive class is *n*_*positive*_, while the number of images from the negative class is *n*_*negative*_ (*n* = *n*_*positive*_ + *n*_*negative*_).

### Image descriptors

In order to be able to classify mammogram images, a classifier must receive relevant characteristics of the images as input data. Medical images are usually large and contain extremely high numbers of pixels; using an algorithm which receives all the image pixels as input data is impractical and can lead to complex programs which require high training times. Consequently, a smaller number of image attributes are extracted based on the position and intensity of certain pixels, through the use of image descriptors.

Image descriptors are divided into three main categories: texture descriptors, colour descriptors and shape descriptors. Since the purpose of our chosen application is to determine only whether or not a mammogram presents cancer, only texture and colour descriptors have been used. Further approaches can involve a more in–depth analysis of mammograms on the basis of tumour contours, where shape descriptors come into their own.

By way of image intensity measures, we make use of moments, histograms of oriented gradients and kernel descriptors.

#### Moments

The intensity of an image pixels give several statistical measures known as moments, from which we include in our analysis the following: mean value, standard deviation, skewness, kurtosis, and the minimum and the maximum intensity values. This combination of statistical measures has been successfully used before for breast cancer diagnosis in [3].

#### Histograms of Oriented Gradients

The computation of the Histograms of Oriented Gradients (HOG) is based on the fact that there is a direct connection between the appearance and the shape of an object and the distribution of intensity gradients.

Using this descriptor for object detection involves the number of gradient orientations in specific regions of the image [15]. Therefore, 1D HOG is computed for small portions (cells) whose union represents the whole image.

When computing the histogram, we start with gamma and colour normalization. A number of mask filters are afterwards applied in order to compute the gradient magnitude. The orientation (the direction of the fastest gradient change) is then computed, which gives an *n* × *m* matrix, where *n* and *m* represent the image size. The HOG is computed based on this matrix, by computing for each cell how many pixels there are where the gradient orientation falls in that specific cell. L1 and L2 norms [15] can be used to normalize the histogram with the aim of obtaining invariance to illumination and shadowing.

#### Kernel descriptors (KD)

The third and final image representation considered is based on the more generic measure of kernel descriptors (KD) [16]. Histogram–based features can be expressed as special cases of efficiently matched KDs.

Due to the fact that the gradient computation takes into account the pixels features of an image patch, the dimensions of the patch need to be known *a priori* in order to be able to compute the gradient orientation. Any similarity measure between two patches can only be computed afterwards. It is this similarity which is taken into account by learning algorithms. When considering the HOG descriptors, this value of similarity is based on the histograms associated with discretized pixels features. The discretization step could lead to quantization errors, limiting the performance of the recognition.

KDs mitigate the discretization disadvantage by using a kernel function for computing the similarity of two patches. This function is a match kernel and is mathematically a Gaussian kernel function.

Named KDs are: the gradient match kernel (considers the magnitude, the orientation and the position and is suitable for capturing the variation of images), the colour kernel (considers the colour and the positions and is suitable for describing the appearance of an image), and the local binary pattern kernel (considers the standard deviation of pixels in the neighbourhood, the differences between the values of the binarized pixels and the position and is suitable for local shape capturing).

KDs convert the features of the pixels into features at the level of the patch, without having to discretize them. The measure of regions similarity is thus based on a match kernel function. Starting from these match kernels, some compact KDs with low dimensionality can be derived using kernel principal component analysis (KPCA) [17].

One level up of abstraction and KDs can also be considered as being applied on sets of KDs. KDs can be applied not only over sets of pixels, but also over sets of KDs. In this hierarchical approach [16, 18], KDs are applied recursively until features emerge from the image.

### Datasets

The datasets used in this study are all publicly available. Each of them has had specific image descriptors applied as per Section.

The Mammography Image Analysis Society (MIAS) [19] is available at: http://www.wiau.man.ac.uk/services/MIAS/MIASweb.html. It offers a set of images along with information such as class of abnormality, severity, location and size.

The Breast Cancer Digital Repository (BCDR) [3], available at http://bcdr.inegi.up.pt/, contains labelled mammographies and ultrasound images and is divided into two smaller datasets: a Film Mammography-based Repository (BCDR-FM) and a Full Field Digital Mammography-based Repository (BCDR-DM). Further details are available at http://bcdr.inegi.up.pt/ and in [3].

The Digital Database for Screening Mammography (DDSM) [20] contains images of breast, patient and image information from around 2,500 studies. The target of these studies was the development of screening research as well as the diagnosis improvement.

**Fig 1:**
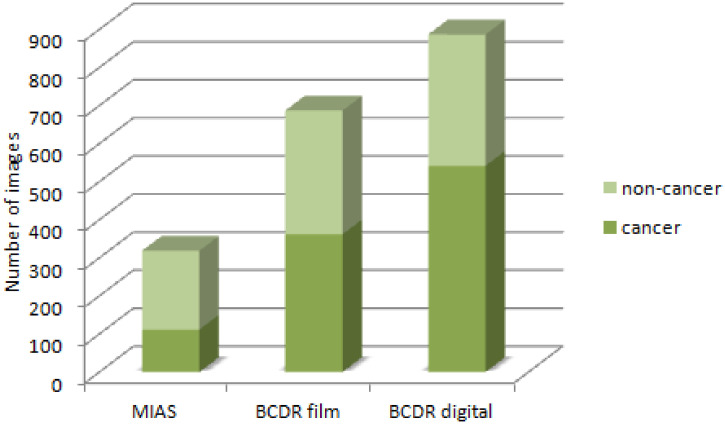
Number of images from patients with and without cancer, from the three different datasets.

Table 1 contains, for each dataset, the total number of images (the second column). Some of the images come from patients with no cancer, and the rest come from patients with cancer. Fig 1 illustrates the same three datasets.

**Table 1:** Dataset distribution.

Set of images	Total	Non-cancer	Cancer			
			calcification	masses	benign	malign.
MIAS	319	207	22	90	64	48
BCDR film	687	325	26	336	187	175
BCDR dig	887	345	201	341	465	77
DDSM	1000	596	89	118	248	156

The considered classifiers are trained on two thirds of the data and tested on the remaining third.

OpenCV http://opencv.org/ has been used for computing the HOG descriptor for each of the three datasets. The used parameters are the following: 720 × 1152 pixels window; 16 × 16 pixels block; 8 × 8 pixels cell; 2 × 2 pixels block stride; 8 gradient bins. A number of 458, 172 features have been obtained for each set of images, all of them being re-dimensioned to 720 × 1152 pixels.

The values used for the KD parameters are the following: kdesdim (which impacts how many features are extracted from a patch) = 200, contrast (which is used by the gradient kernel) = 0.8, grid size = 8 and patch size = 16, resulting in a number of 10, 500 features for each set of images. These values are actually those proposed by the authors of KD.

It is well known that the training data strongly influences the classifier’s performance. An analysis of the complexity of our data is presented in Section, so as to relate the data to the classifier behaviour.

### Methods

We illustrate this application with Multi–Expression Programming (MEP) [21], by proposing a multi–objective (MO) version based on NSGA-II [22], which is known due to its rapid convergence and ability to preserve the diversity in the space of solutions.

Being a linear flavour of Genetic programming (GP) [23], MEP is able of automatically discovering optimal calssifiers using techniques inspired by the Darwinian evolution principle. A population of classifiers, also called chromosomes, gradually evolves during a number of generations, using bio-inspired mechanisms like the natural selection, mutation and crossover [23]. Out of the advantages of MEP, we remember its flexibility and its ability not only to represent data, but also to perform computations and to pre-process and post-process data. More precisely, MEP is able to automatically transform the input data by applying different methods, like the selection of subsets of features, the creation of new characteristics by applying certain functions on the original attributes, the assessment of the relevance or importance of certain features, etc. Moreover, MEP is able to solve both linear and non-linear classification problems, without specifying the type of the problem apriori. Discriminant functions evolved by the MEP algorithm are similar to the mathematical operations and transformations used in image processing, thus MEP-based models are considered to be very suitable for image classification tasks [10].

**Fig 2:**
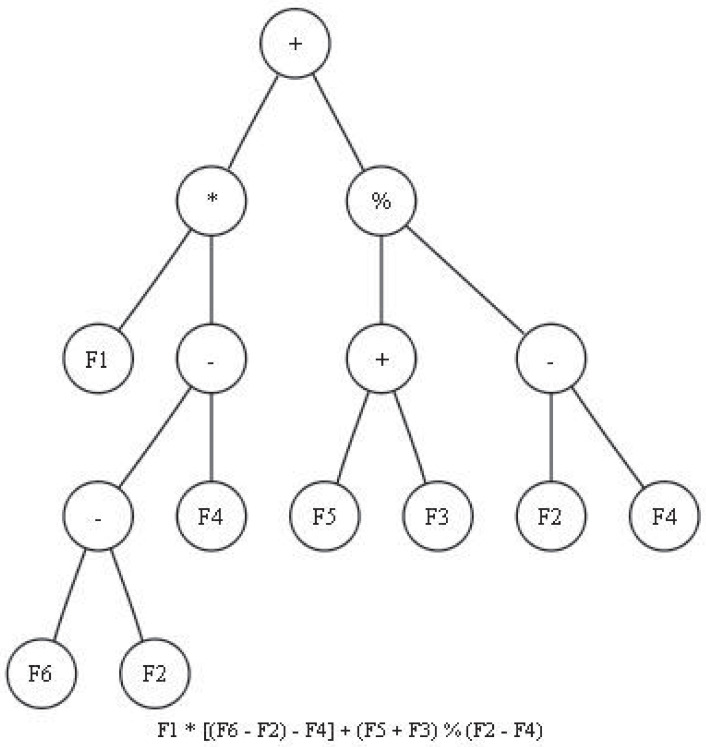
MEP-based classifier example.

The chromosomes involved in a MEP algorithm can be viewed as trees with leaves (that consist of image features and constants) and internal nodes (that consist of various arithmetic operators). For instance, if we suppose a classification model based on statistical moments associated to a given image, the terminal set will be composed by six elements: *TS* = *F*_1_, *F*_2_, *F*_3_, *F*_4_, *F*_5_, *F*_6_, where *F*_1_ represents the mean, *F*_2_ the standard deviation, *F*_3_ the skewness, *F*_4_ the kurtosis, *F*_5_ the minimum and *F*_6_ the maximum intensity values of an input image. A possible classifier based on this terminal set and on the above mentioned function set *FS* is given in Fig 2.

The fitness function shows the goodness of a solution and its performance in solving a given problem [23]. Solutions with better fitness have higher chances of surviving to the next generation and, thus, of propagating their genetic material, while solutions with low fitness functions are gradually removed from the population.

In the canonical single-objective approach, a single individual, which is the fittest one, is returned at the end of the training process. However, this method can be extended by using the multi-objective optimization, an adaptation of the learning process that considers multiple, conflicting objectives, treated separately in the optimization. A set of best trade-off solutions, also called Pareto front, is then simultaneously evolved along the objectives in a single optimization run, allowing the end-user to choose the preferred classifier a posteriori.

In order to achieve this, two modifications have to be performed on the canonical single objective approach. First of all, the evolutionary search algorithm has to be adapted to simultaneously evolve a set of genetic program solutions along the learning objectives. The solutions of this set form the Pareto front. Then, the fitness function needs to be modified to use the Pareto dominance, by ranking the solutions according to their performance on all the learning objectives relative to all other solutions in the population. More specifically, a solution *S*_*i*_ is said to *dominate* another solution *S*_*j*_ if *S*_*i*_ is at least as good as *S*_*j*_ on all of the objectives, and better on at least one, while a solution is called *non-dominated* if it is not dominated by any of the solutions from the population. The set of non-dominated individuals is improved over a number of generations, by combining parent and offspring populations, and by selecting the fittest individuals from this merged population as the parent population for the next generation. At the end of the evolutionary search, the algorithm will return the individuals from the Pareto front, more specifically, all of the non-dominated solutions from the population.

This paper uses as a multi-objective optimization algorithm the well established NSGAII [24]. In this approach, the fitness value for a solution *S*_*i*_ is represented by its dominance rank, defined as the number of other solutions from the population that dominate *S*_*i*_. Therefore fitness values in NSGAII need to be minimized: non-dominated solutions have the best fitness of 0, while solutions with poor performances, which are dominated by many individuals, have high fitness values. In addition to the Pareto dominance, a second fitness measure, called crowding distance, is used to ensure a good spread-off of solutions across the trade-off frontier. Thus, when two or more individuals from the population have the same Pareto ranking, the one from the most sparsely populated objective region will be preferred over the ones from densely populated regions. More specifically, the crowding distance for a specific solution represents the average Manhattan distance to the solution’s nearest neighbors along each of the objectives.

Each MEP chromosome encodes multiple solutions (in our case, multiple classifiers). In order to identify the best classifier encoded into a chromosome, a quality measure *X* is required. In order to determine the fitness of an MEP chromosome (this approach being MO), more objectives expressed by quality measures *Y* are used. The measures *Y* are computed by taking into account only the best classifier encoded into the current chromosome. In order to compare the performance of the classifiers obtained in this way on our chosen datasets, we need a set of performance measures *Z*. These are described in Section. Taking into account all these aspects, a classifier will be denoted by indicating the *X* and *Y* measures.

When evaluating a classifier encoded into an MEP chromosome for a specific datapoint, a single value is computed, which indicates its predicted class. When there are two output classes, one can use a threshold for mapping the resulting numeric values to the binary output values. Two aspects should be considered related to this threshold:

- its value range: the output remains within [0, 1] due to a sigmoid function used for the resulting value; threshold values from [0, 1] are used for performing the mapping to the binary output values;
- its optimal value: we carry out an optimisation of the area under the curve (AUC); the corresponding set of thresholds is analysed in order to find the sub-classifier with the best results; this is known as AUC optimisation [25]. We also discuss the extent to which this measure is a good choice for determining the best sub–expression from an MEP–based classifier.

#### Quality measures for a classifier

When a classifier has been evolved through an evolutionary algorithm, different objectives have been taken into account:

- the classification error rate (as an opposite of accuracy, computed by dividing the number of instances that have been correctly classified to the total number of instances—see Eq 1) and the computational cost [26]
- accuracy of the minority (positive) class (true positive rate or sensitivity—see Eq 2) and accuracy of the majority (negative) class (true negative rate or specificity—see Eq 3) [27, 28]
- values from a histogram associated with the ROC [29]. A new way of representing the ROC curve is considered, as a histogram of TP rates against FP rates, which are fixed. For each bin of the histogram, the mean of the TP rates contained within is computed.

$$\begin{array}{ccc} Acc & = & \frac{\text{correctly}\;\text{classified}\;\text{items}}{\text{all}\;\text{items}}=\frac{TP+TN}{TP+FP+TN+FN} \end{array}$$ (1)

$$\begin{array}{ccc} Acc_{min} & = & Acc_{pos}=\frac{TP}{TP+FN} \end{array}$$ (2)

$$\begin{array}{ccc} Acc_{maj} & = & Acc_{neg}=\frac{TN}{TN+FP} \end{array}$$ (3)

Regarding the *X* measures involved in our approach, since an MEP chromosome is able to encode more sub-expressions (in fact, more classifiers) and the best one of them has to be selected,

- the average accuracy over the considered thresholds (denoted *AvgAcc*),
- the AUC associated to the considered thresholds (denoted *AUC*),
- the geometrical mean (GM) of positive accuracy and negative accuracy for a single threshold (denoted *GM*) or
- the average of GMs between positive accuracy and negative accuracy over more thresholds (denoted *avgGM*)

is used in order to select the best sub-classifier.

Regarding the *Y* measure, our MO approach takes into account the following objectives:

- the classification accuracies computed for pre-established thresholds (denoted *Accs*);
- values from the histogram (each bin averages all TPs from that bin) of TP/FN rates (for pre-established) thresholds (denoted *HistoTP*/*FNrates*);
- the positive and the negative class accuracies for a single threshold (Denoted *PosNegAcc*);
- the geometrical mean of the positive and the negative class accuracies for pre-established thresholds (Denoted *GMPosNegAcc*.

In our approachm, the model of dominance between two classifiers proposed in [29] is considered. This is a problem that has to be discussed when working with multi-objectives techniques.

#### Classifier comparison methods

In multi–objective optimization, a Pareto front of optimal solutions is generated. It is therefore necessary to decide which classifier or which combination of classifiers should be used. There are several methods to deal with classifier ensembles (majority voting, linear combination of classifiers from the Pareto front, a meta-classifier over the simple classifiers from the Pareto front). In our own classification model we make use of the popular majority voting scheme. This means that each classifier gets applied to decide the class of a certain instance, and the class chosen eventually is the one that has been predicted by the majority of the classifiers. This straightforward approach together with other voting schemes are widely used in the literature [11, 28, 30–35].

Of the classifiers in the final Pareto front obtained at the end of the training stage, only one will be used for the testing stage. The predicted class will be chosen from all classifiers of the final front so as to represent the answer given by most of the classifiers for each new datapoint.

Let us suppose that the training process gives a front of *r* classifiers (*c*^1^, *c*^2^, …, *c*^*r*^) in one run of MO-MEP algorithm. Several medical images are used for testing the classifiers (*TS*—testing size). The class of each testing image is determined in the following way:

- Every classifier *c*^*i*^, with *i* = 1, 2, …, *r*, predicts which is the class associated with each image *im*_*j*_, with *j* = 1, 2, …, *TS* from the testing set;
- The predicted classes form a matrix of labels *LM* with *r* lines and *TS* columns;
- The label (e.g. truth value) decided by the majority of classifiers is associated with each image in the testing set.
  Dealing with a binary classification problem, one can use the majority function from Boolean algebra or the median operator. Let us consider the case where an image can have one of the following two labels: *True* (*T* indicates the presence of pathologies) and *False* (*F* indicates a clear scan). In our case, we have to map *r* possible inputs for each image (representing all possible labels) into one label. If there are more than *r*/2 inputs having the value *False*, the function will be evaluated to *False*. Otherwise, the function will be evaluated to *True*. Eq 4 can be used to determine the majority label, where *True* is represented as 1 and *False* is represented as 0. In this equation the brackets represent the greatest integer function.
  $$\begin{array}{c} label_{im_{j}}=Majority\left({LM}_{j,1},\ldots ,{LM}_{j,r}\right)=\lfloor \frac{1}{2}+\frac{(\sum_{i=1}^{r}{LM}_{j,i})-\frac{1}{2}}{r}\rfloor \end{array}$$ (4)

#### Performance measures for the classifiers

Different criteria have been considered in order to evaluate the performance of the classifiers on our chosen datasets:

- the computational complexity of evolving a classifier and the obtained classification error (the accuracy) [26]
- diversity (the average number of different Pareto front solutions) and hyperarea (the area under the Pareto front) [27]
- the accuracy of the minority class and of the majority class respectively, together with the average size of the evolved ensembles [32]
- AUC [29]
- average hyperarea of the evolved fronts (Pareto–approximated and Pareto–optimal) [28].

#### Combining them into MO models

From the many models used in the literature, we investigate the use of the following four approaches. We distinguish betweeen them mainly in the objectives considered in each case. Notation *X*&*Y* stands for a classifier that uses metric *X* to select the best sub-expression encoded into an MEP chromosome and metric *Y* as multi-objective optimisation.

- Model A: AvgAcc & Accs—the average accuracy over pre-established thresholds indicates the best sub-expression of an MEP chromosome and the individual accuracies (for each of these pre-established thresholds) represent the optimisation objectives;
- Model B: AUC & histoTP/FN rates—the AUC over pre-established thresholds indicates the best sub-expression of an MEP chromosome and the histogram (each bin averages all TPs from that bin) of TP/FN rates (for pre-established) thresholds indicates the optimisation objectives;
- Model C: GM & PosNegAcc—the geoemtric mean of positive and negative accuracy indicates the best sub-expression of an MEP chromosome and two optimisation objectives are considered: the positive and the negative class accuracies for a single threshold;
- Model D: avgGM & GMPosNegAccs—the optimisation objectives are the geometrical mean of the positive and the negative class accuracies for pre-established thresholds, while the best MEP-subexpression is selected by taking into account the average of these objectives.

The results contain two categories of Accuracy and AUC, respectively, all computed on the testing data and associated to *Z* measure.

- the average values of Accuracy/AUC computed on the test sets in several runs of the algorithm—the training and testing take place in each algorithm run; the voting scheme is afterwards applied to all classifiers that belong to the optimal Pareto front, considering all examined threshold values.
- the general optimal values of Accuracy/AUC computed on the test sets after all the runs are executed—the general Pareto front represents the set of all classifiers that belong to the Pareto front, in each algorithm run; the voting scheme is afterwards applied to all classifiers that belong to the general optimal Pareto front, considering all examined threshold values, and the general optimal values for Accuracy/AUC can therefore be computed.

Note that in the case of model C a single threshold in involved in the process of classifier’s quality evaluation and the AUC-based performance is not computed, being irrelevant.

### Data complexity measures

This section presents a brief characterisation of our data complexity, trying to relate it to the classifier behaviour. We investigate several measures which characterise the difficulty of a classification problem (borrowed from both supervised and unsupervised learning) that are independent of the used learning algorithm, since, in the case of discriminant classifiers, the cluster structures which emerge in the feature space may help (or hinder) the learning process. Furthermore, it was already proven [36] that real–world problems (such that those considered in our numerical experiments) contain structures in the measurement space that are significantly different from the theoretical problems (constructed by a random labeling of points).

We have considered the following measures [36]:

- Fisher’s Discriminant Ratio (*F*_1_)—it computes the discriminating power of a feature, identifying the overlap among different classes by considering the distribution of values with respect to each feature; this measure emphasizes the geometrical characteristics of the class distributions;
- Separability of classes (*N*_2_)—compares the spread inside the classes and the distance between the classes, being aimed to evaluate the separability of classes by analysing the shape of their boundaries.
- Average number of points per dimension (*T*_2_)—it measures the shapes of each class manifolds.

These three complexity measures have been applied to the breast-cancer problems described in Section for all image representation (for both parts of a dataset: training and testing). The corresponding obtained values of these measures are given in Table 2.

**Table 2:** Fisher’s discriminant, N2 and T2 measures for all problems (and representations).

Database	Represen-tation	F1↑		N2↓		T2↑	
		train	test	train	test	train	test
MIAS	Mom	0.0029	0.0056	2.3853	1.2526	30.2857	15.2857
	HOG	0.0047	0.0076	0.9944	0.9927	0.0005	0.0002
	KD	0.0067	0.0231	1.2318	1.2739	0.0202	0.0102
BCDR film	Mom	0.0082	0.0016	1.0742	1.7043	65.2857	32.8571
	HOG	0.0026	0.0044	0.9786	0.9956	0.0010	0.0005
	KD	0.0002	0.0181	1.0287	0.0487	0.0435	0.0219
BCDR digital	Mom	0.0013	0.0195	0.8139	1.0944	84.4286	42.2857
	HOG	0.0029	0.0167	0.8684	0.8719	0.0013	0.0006
	KD	0.0005	0.0011	0.8647	0.9763	0.0563	0.0282
DDSM	Mom	0.0003	0.0132	5.7287	0.0006	76.4286	38.2857
	HOG	0.0057	0.0114	0.9049	0.3867	0.0012	0.0006
	KD	0.0384	0.0384	0.8296	0.8296	0.0510	0.0255

The values corresponding to these complexity measures indicate that:

- if we consider the training stage and
  - F1 measure, the DDSM+KD problem is the simplest one and the BCDR film + KD is the most onerous one,
  - N2 measure, the BCDR digital + Mom problem is the simplest one and the the DDSM + Mom is the most onerous one,
  - T2 measure, the BCDR digital + Mom problem is the simplest one and the MIAS + HOG is the most onerous one;
- if we consider the testing stage
  - F1 measure, the DDSM + KD problem is the simplest one and the BCDR digital + KD is the most onerous one,
  - N2 measure, the DDSM + Mom problem is the simplest one and the BCDR film + Mom is the most onerous one,
  - T2 measure, the BCDR digital + Mom problem is the simplest one and the MIAS + HOG problem is the most onerous one.

It follows that a problem can be classed as onerous using one measure, but at the same time, another measure can class the same problem as simple. This observation is consistent with each measure capturing different aspects of the classification results.

## Results and discussion

### Numerical results

Before to investigate the sensitivity of the classification performance to the optimisation criteria, a preliminary experiment was dedicated for comparing two basic strategies for combining the classifiers: majority voting and simultaneous multi-objective optimisation. In the majority voting approach, more classifiers (with a single optimisation criterion) are applied in order to decide the class of a certain instance, and the chosen class is the one that has been obtained by the majority of the classifiers. This very simple and straightforward approach together with other voting schema are widely used in the literature [31, 32, 35]. In simultaneous multi-objective optimization, an adaptation of the learning process that considers multiple, conflicting objectives, are treated separately in the optimization. A set of best trade-off solutions (forming the Pareto front) is then simultaneously evolved along the objectives in a single optimization run, allowing the end-user to choose the preferred classifier a posteriori. In both scenarios (voting and simultaneuos optimisation) the same objectives have been considered. We compared the performances by involving the objectives of model A—the average accuracy over pre-established thresholds indicates the best sub-expression of an MEP chromosome and the individual accuracies (for each of these pre-established thresholds). The classifier’s inputs have been fixed to statistical moments. The results obtained in this experiment (see Table 3) indicate the potential of the simultaneous multi-objective approach.

**Table 3:** Comparison of voting and simultaneous MO strategies.

Database	Voting	MO
MIAS	60.07	61.28
BCDR film	83.03	92.00
BCDR digital	44.18	48.86
DDSM	45.64	50.41

The second aim in our investigation was to analyse different MO scenarios of the considered classifiers. We have investigated the performance of the evolved MO classifiers on the test datasets. Numerical results are given in Tables 4–7. In Tables 4 and 5, the average AUC and accuracy, respectively, computed during testing stage over more runs are presented for all models (A, B, C, D) and all image representations (Mom, HOG, KD). In each run of the learning algorithm, the training and testing stages are performed; the trained classifiers belonging to the optimal Pareto front are tested by using the voting procedure.

**Table 4:** Average AUC.

Database	Representation	A	B	D
MIAS	Mom	10.44	46.89	9.28
	HOG	32.93	50.00	12.46
	KD	0.06	33.33	23.26
BCDR film	Mom	21.46	50.00	13.72
	HOG	23.28	51.39	17.26
	KD	50.35	51.14	49.46
BCDR digital	Mom	37.24	50.00	27.07
	HOG	35.21	50.00	27.87
	KD	49.60	50.31	27.35
DDSM	Mom	3.54	50.00	12.41
	HOG	26.16	49.94	16.98
	KD	0.12	65.22	20.14

**Table 5:** Average Acc.

Database	Representation	A	B	C	D
MIAS	Mom	61.28	49.16	41.74	54.89
	HOG	51.00	52.90	61.99	52.96
	KD	63.93	45.17	46.73	53.05
BCDR film	Mom	46.86	49.48	53.19	49.77
	HOG	47.68	49.68	52.75	49.74
	KD	49.87	49.71	50.43	49.97
BCDR digital	Mom	50.41	47.77	51.69	49.16
	HOG	47.24	47.77	41.78	50.63
	KD	58.94	49.12	51.24	49.35
DDSM	Mom	9.20	40.15	63.68	38.59
	HOG	37.89	40.19	33.71	45.34
	KD	75.23	50.16	48.47	62.05

**Table 6:** AUC Pareto optimal front.

Database	Representation	A	B	D
MIAS	Mom	1.37	47.56	11.71
	HOG	37.51	50.00	27.71
	KD	0.17	52.96	37.55
BCDR film	Mom	31.66	50.00	18.65
	HOG	33.59	43.10	31.50
	KD	48.64	48.64	48.64
BCDR digital	Mom	38.25	50.00	26.17
	HOG	48.24	50.00	40.33
	KD	58.92	51.58	49.53
DDSM	Mom	0.00	50.00	21.52
	HOG	31.39	49.62	22.18
	KD	0.05	57.88	27.83

**Table 7:** Acc Pareto optimal front.

Database	Representation	A	B	C	D
MIAS	Mom	64.30	45.23	52.34	51.22
	HOG	52.52	52.90	61.99	47.48
	KD	64.49	50.00	42.06	47.20
BCDR film	Mom	46.74	49.48	52.61	51.83
	HOG	50.78	51.65	53.91	48.83
	KD	50.13	50.13	54.35	50.13
BCDR digital	Mom	51.28	47.77	57.77	47.80
	HOG	50.54	47.77	54.05	50.00
	KD	58.92	48.68	62.84	49.53
DDSM	Mom	8.40	40.15	35.82	40.82
	HOG	28.58	41.94	57.09	46.75
	KD	74.50	50.37	32.34	57.68

In Tables 6 and 7 the general optimal values for AUC and Accuracy, repectively, computed on the test sets after all the runs are given. The general Pareto front represents the set of all classifiers that belong to the Pareto front in each algorithm run; the voting scheme is then applied to all classifiers that belong to the general optimal Pareto front, considering all examined threshold values, and the general optimal values for AUC/Accuracy can be computed.

The values from Tables 4 and 5 indicate that, by considering Average AUC objectives, model B performs better for all databases and for all image representation, but when the Average Acc objectives are considered (see Tables 6 and 7), the model C performs better. Sometimes, model A was the best classifier. In this context, a statistical analysis of the obtained results must be performed in order to increase the generality of such conclusions.

### Statistical analysis methods

Estimating the classification accuracy measurements of each classifier presented is no small feat. The multi–objective approach means that each classifier converges over multiple values of its Pareto front. Even comparisons between pairs of classifiers require multiple comparisons, involving multiple algorithm pairings over multiple problem domains. The pros and cons of each of the classifiers presented make it impossible to choose a single scheme over the rest.

Numerical values specific to each test are computed for each pair of classifiers in a variety of contexts. These values make sense within the parameterisation of each test but cannot be compared between different statistical tests.

In order to compare the relative merits of the classifiers under different tests, as well as to determine the statistical significance of the results, we also calculate the corresponding *p*–value. This number between 0 and 1 can be used to accept or reject the Null Hypothesis. A significance level *α* is chosen for this purpose. The comparison *p* < *α* indicates strong evidence against the Null Hypothesis, meaning that the test is unsuitable for comparing those two classifiers.

The tool selection and its parameters are based on conventional choices in the literature [37]. As such, throughout this paper we set the statistical confidence level to 95%, which corresponds to a significance level *α* = 0.05.

Whenever *p* < *α* values of *p* are shown in boldface.

We present a range of ranking tools for comparing classifiers, highlighting the disagreements between such comparisons and concluding that the comparison tools themselves bias for or against specific schemes.

#### Wins, ties and losses

**Fig 3:**
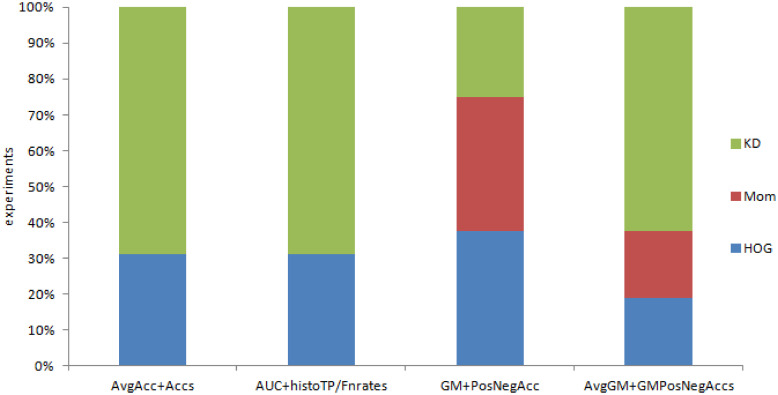
Performance of representations based on Moments (Mom), Histogram of Oriented Gradients (HOG), Kernel Descriptors (KD) as percentage of wins, ties and losses (for all classification models).

**Fig 4:**
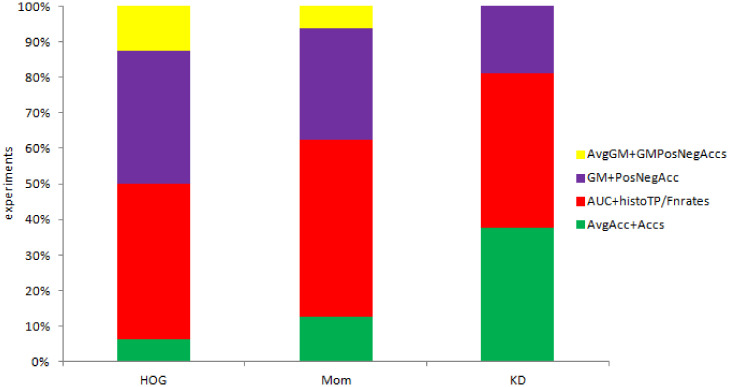
Performance of classification models (A, B, C, D) for all image representations as percentage of wins, ties and losses (for all classification models).

One of the most simplistic estimates, the *wins, ties and losses* method offers a single numerical estimate of win percentage, in that it compares the number of datasets over which an algorithm is the overall winner. This can be used to compare either the win percentage of an image representation over other representations (as per Fig 3), or the win percentage of a classification method against other methods (as per Fig 4).

The comparison of the three image representations (Moments, HOG and KD) across the four classificaton models indicates that the best representation for three classification models is KD, while the win for best representation under one model (C) is shared by HOG and Mom. This is shown in Fig 3. Fig 4 turns the comparison the other way around, comparing the four classification methods. The AUC&histoTP/FPrates model wins across the three image representations.

#### Sign test

The Sign Test is a significance test between the number of winnings and losses, while the Wilcoxon’s Signed Ranks Test is a significance test using both the number of winnings and looses and the level of winnings/losses (difference between actual two data).

The performance of classification algorithms based on machine learning is often evaluated using the *paired t–test*. Since this is a parametric analysis and requires conditions such as independence, normality, heteroscedasticity [38], it has been impractical to use it to compare our classifiers. Instead, we have opted strictly for non–parametric (distribution–free) tests, such as the *Sign test* and the Wilcoxon signed rankings for comparing two classifiers, as well as the Friedman test with the related post–hoc tests for comparing several classifiers over different data sets.

Our statistical analysis is a post–hoc procedure aiming to highlight the disagreements between the various classifiers.

The Sign test compares pairs of classification models by taking into account their number of wins, ties and losses over all image representations.

The Sign test is a classic form of inferential statistics and the null hypothesis assumes that, if two algorithms are compared, each should win approximately $nc/2+Z\sqrt{(}nc)/2$ out of nc datasets or problems.

In Eq 5, *nc* is the total number of cases, out of which we single out the number of wins: $nc_{w}\left(A↔B\right)$ represents the number of cases when model A performs better or the same to model B; *Z*_*α*_ is the value that corresponds to the *z* statistic at significance level *α*. The null hypothesis, that classification model A and classification model B perform equally well, is rejected if the inequality in Eq 5 is satisfied. The critical *p*–values corresponding to *nc*_*w*_ have been inferred from statistical tables.

$$\begin{array}{ccc} & & nc_{w}\left(A↔B\right)\geq \frac{nc}{2}+Z_{\alpha }\frac{\sqrt{nc}}{2} \end{array}$$ (5)

The results of applying this test over all non–trivial pairs of classification models are presented in the triangular Table 8.

**Table 8:** Results of sign test: $nc_{w}\left(A↔B\right)$ for all possible combinations and the corresponding p–value. *nc* = 4*databases* × 3*representations* = 12.

	A	B	C	D
A	—	15 (p = 0.999)	**33 (p = 0.009)**	**31 (p = 0.052)**
B		—	**31 (p = 0.030)**	29 (p = 0.151)
C			—	16 (p = 0.993)
D				—

For our chosen significance level *α* = 0.05 we get *Z*_*α*_ = 1.645, and hence the null hypothesis is *accepted* for pairings:

- model A vs. model B,
- model B vs. model D, and
- model C vs. model D.

The null hypothesis is rejected for the remaining pairs. Therefore, on the basis of the sign test only, we can say that (*p* < 0.05):

- model A outperforms model C,
- model A outperforms model D, and
- model B outperforms model C.

The sign test does not take into account the magnitude of the differences between the compared algorithm’s performances. Furthermore, the null-hypothesis of this test is rejected only one algorithm almost always outperforms the other algorithm.

#### Wilcoxon test

The Wilcoxon sign ranking test [39] is more sensitive than the Sign test due to taking into account both the number of wins and losses and their level. A ranking is then carried out based on the absolute value of the difference in performance of two algorithms over each of the data sets.

The Wilcoxon ranking is a non-parametric counterpart to the paired t–test, it goes beyond normal distributions and is not affected by the presence of outliers.

If we denote by diff_*i*_ the performance difference of the two algorithms A and B on the *i*^*th*^ out of *n* data sets, then the sum of ranks for those data sets where B outperformed A is computed by Eq 6 and for the opposite is computed by Eq 7. The smaller of the two sums is *T* = *min*(*R*^+^, *R*^−^). Since in our case the number of datasets is way smaller than 25, the precise critical values for T can be found in statistical textbooks.

$$\begin{array}{ccc} R^{+} & = & \sum_{{\text{diff}}_{i}>0}rank\left({\text{diff}}_{i}\right)+\frac{1}{2}\sum_{{\text{diff}}_{i}=0}rank\left({\text{diff}}_{i}\right) \end{array}$$ (6)

$$\begin{array}{ccc} R^{-} & = & \sum_{{\text{diff}}_{i}<0}rank\left({\text{diff}}_{i}\right)+\frac{1}{2}\sum_{{\text{diff}}_{i}=0}rank\left({\text{diff}}_{i}\right) \end{array}$$ (7)

An ideal goal would be to reject the null hypothesis (that two algorithms perform equally well). To this end, we computed the Wilcoxon test for all pairs of considered classification models considering all four performance measures (for each descriptor and for all descriptors, respectively).

**Fig 5:**
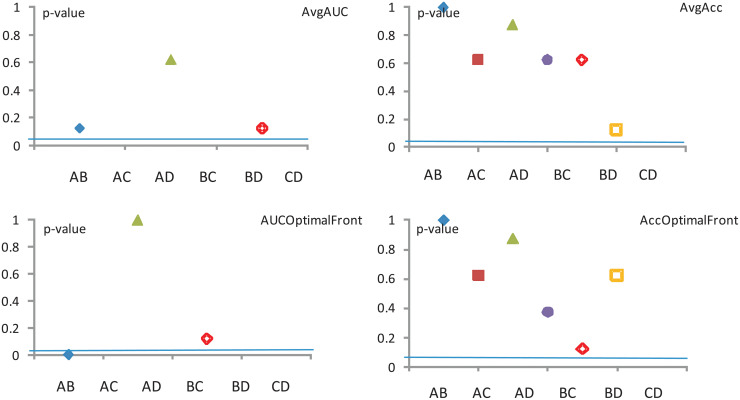
New Wilcoxon test—Mom. The significance level is *α* = 0.5.

**Fig 6:**
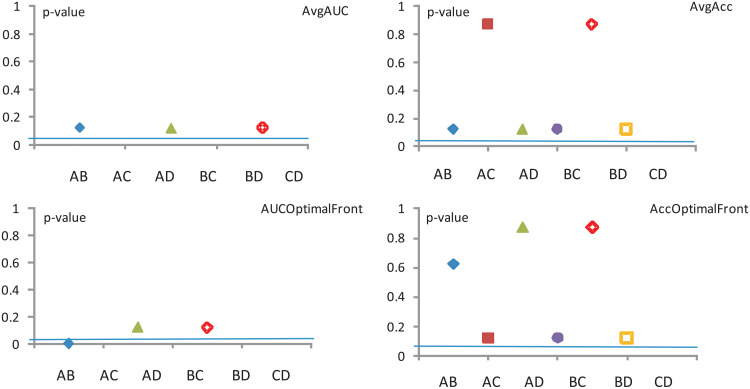
Wilcoxon test—HOG. The significance level is *α* = 0.5.

**Fig 7:**
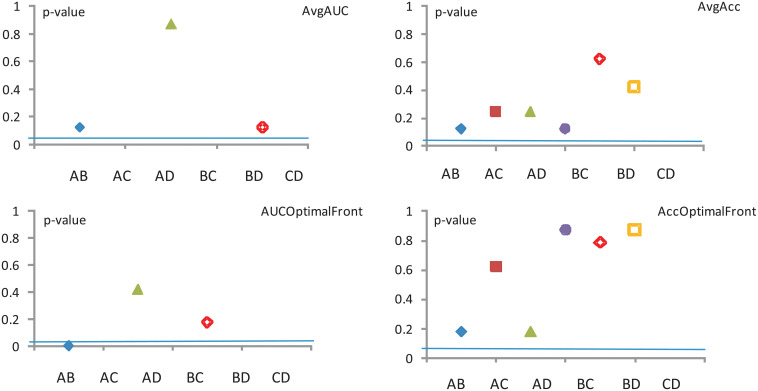
Wilcoxon test—KD. The significance level is *α* = 0.5.

The results of these tests are compiled in Figs 5–7. The corresponding *p*–values are provided in brackets. Like before, we choose a significance level *α* = 0.05 and *N* = 4 data sets. Considering the exact critical values for the Wilcoxon’s test, for these *α* and *N* values, the disagreement between the classifiers is statistically significant if the test’s *z* value is smaller than −1.96 or *p* < *α*. Unfortunately in all of our comparisons using the three individual data representations (HOG, Mom and KD) the null hypothesis is *not* rejected, which indicates that the Wilcoxon test is insufficient by itself, and a more sensitive tool needs to be used. Sections—detail other such tests.

Note that, since Model Conly feature an Acc, there are no corresponding values (i.e. the third column is missing) in all of the tables which deal with an Avg AUC calculation.

Collating the above information over the same datasets regardless of image representation, we can draw conclusions about the four performance measures used.

1. The assessment of the **Avg AUC** performance measure across all three image representations reveals that (*p* < 0.05)
  - model A significantly outperforms model B,
  - model B significantly outperforms models C and D, and
  - model C significantly outperforms model D.
  Meanwhile, for other pairings (e.g. model Avs. model D) there is no detectable disagreement (*p* > 0.3).
2. The assessment of the **Avg Acc** performance measure across all three image representations reveals that (*p* < 0.05)
  - model B significantly outperforms model D,
  while for other pairings there is no detectable disagreement (*p* > 0.5).
3. The assessment of the **AUC optimal** performance measure across all three image representations reveals that (*p* < 0.05)
  - model A significantly outperforms model B,
  - model B significantly outperforms models C and D, and
  - model C significantly outperforms model D.
  Meanwhile, for other pairings (e.g. model Avs. model D) there is no detectable disagreement (*p* > 0.8).
4. The assessment of the **Acc optimal** performance measure across all three image representations reveals that no evidence of significant disagreements is detected (*p* > 0.2) for any of the pairings.

**Fig 8:**
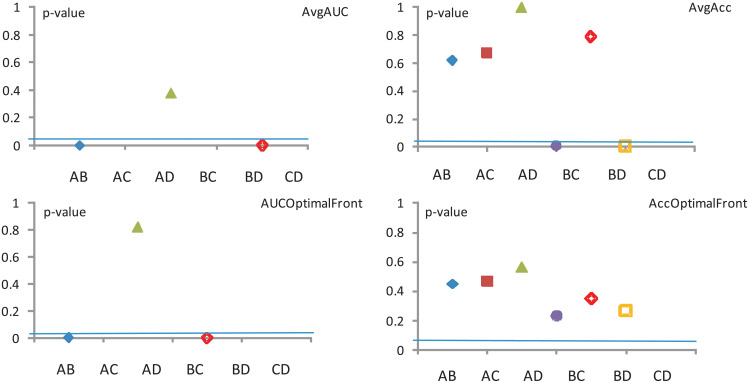
Wilcoxon test—All representations. The significance level is *α* = 0.5.

In conclusion, according to the Wilcoxon test (see results from Fig 8, the models C and D could be discarded altogether. However, these models will be rehabilitated in Sections—where using a different yard stick will give them a higher level or credibility.

#### Friedman

In the case of comparing multiple algorithms over several problem domains, there is a need for more advanced tests. In order to compare one algorithm with other *N* algorithms, as well as all *N* algorithms to each other, a set of post–hoc procedures is needed.

The Friedman test [40] or its related Iman and Davenport test [41] are used in such situations. The main goal of these tests is to perform a ranking of the algorithms. Both tests give information about the existence of disagreements between the result samples compared.

In the case of our data, a comprehensive evaluation considers all the experiments (four classification models) over all three (HOG, Mom and KD) image representations.

The null–hypothesis was reformulated in order to state that the four recognition models perform equally well. The Friedman test was applied in order to establish whether or not to reject this null–hypothesis. In the case of Friedman test, the algorithms are ranked so that the rank 1 is given to the algorithm with the best performance, for each data set. The ranks are averaged in case of ties.

Let us consider $r_{i}^{j}$ being the rank assigned to the *j*-th algorithm on the *i*-th data set, having a total of *k* algorithms and *N*_*ds*_ data sets. The average ranks of all algorithms are computed as $R_{j}=\frac{1}{N_{ds}}\sum_{i=1}^{N_{ds}}r_{i}^{j}$. Under the null-hypothesis, according to which the performances of all algorithms are equal and so their values for *R*_*j*_ should be equal, the Friedman statistic

$$\begin{array}{c} \chi_{F}^{2}=\frac{12N_{ds}}{k(k+1)}[\sum_{j=1}^{k}R_{j}^{2}-\frac{k{\left(k+1\right)}^{2}}{4}] \end{array}$$ (8)

is distributed according to $\chi_{F}^{2}$ with *k* − 1 degrees of freedom.

**Fig 9:**
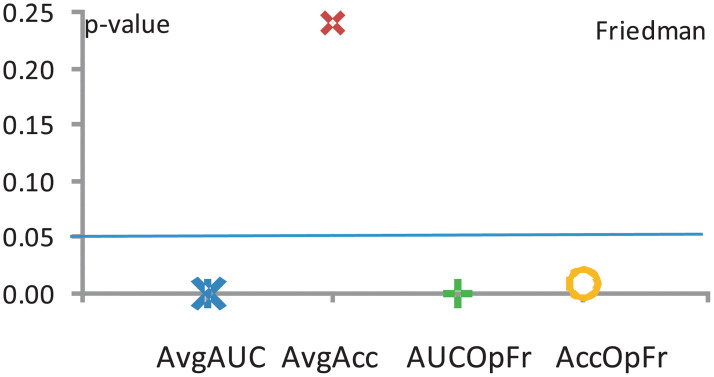
The results of Friedman’s test ($\chi_{t}^{2}$) for all performance measures, degree of freedom 3, the significance level *α* = 0.05 and the critical value *p*_*α*_ = 7.7.

For our specific numbers of algorithms (*k* = 4) and data sets (*N*_*ds*_ = 12 = 4*datasources* × 3*representations*), exact critical values can be computed. The results of applying the Friedman test are shown in Fig 9.

We continue to work with a level of significance of *α* = 0.05. The degree of freedom is 3 due to the fact that we compare four classifiers. We extracted from Distribution Tables [42] the corresponding critical value, which is 7.7.

Therefore, the null–hypothesis is rejected for the first and third performance measures taken into account (A = AvgAcc&Accs and C = GM&PosNegAcc). The Friedman performance measures for Model B = AUC&histoTP/Fnrates and for Model D = avgGM&GMPosNegAccs indicate that there is no significant disagreement between the four considered classification models.

#### Iman and Davenport

In order to identify the existence of disagreements between all result samples compared, it is also possible to apply the Iman—Davenport test [41]. This is a test related to Friedman but less conservative and free of parameters. The expression of Iman—Davenport is given in Eq 9 and it is distributed according to the F-distribution with *k* − 1 and (*k* − 1)(*N*_*ds*_ − 1) degrees of freedom. As before, *k* is the number of algorithms and *N*_*ds*_ is the number of data sets.

$$\begin{array}{c} F_{F}=\frac{\left(N_{ds}-1\right)\chi_{F}^{2}}{N_{ds}\left(k-1\right)-\chi_{F}^{2}} \end{array}$$ (9)

**Fig 10:**
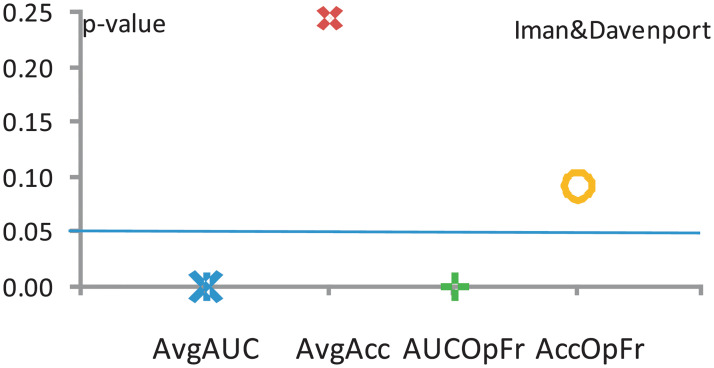
The results of the Iman—Davenport test (*F*_*F*_) for each performance measure, for *k* − 1 = 3 and, respectively (*k* − 1)(*N*_*ds*_ − 1) = 3×11 = 33 degrees of freedom, for significance level *α* = 0.05 and critical value *p*_*α*_ = 2.9223.

Fig 10 shows the Iman—Davenport values (*F*_*F*_). The corresponding Distribution Table [42] critical value for a level of significance *α* = 0.05 is *p*_*α*_ = 2.9223. Since the Iman—Davenport test values are clearly greater than their associated critical value in two cases, it follows that there are significant disagreements between the observed results. A post–hoc statistical analysis is therefore needed in these two cases.

Figs 9 and 10 show the results of applying the Friedman and Iman—Davenport tests. When comparing the statistics of Friedman and Iman—Davenport and their associated critical values, we conclude that there are significant disagreements between the obtained results with a probability error *p* ≤ 0.05. A post–hoc statistical analysis is therefore needed.

The next section illustrates the application of several post-hoc tests over the performances of the considered algorithms. This highlights pairs of algorithms which disagree.

#### Nemenyi

The Friedman and Iman—Davenport tests only show whether or not there is a significant disagreement between the various approaches, but neither of them shows where any discrepancies occur. In cases where the null–hypothesis is rejected, additional post–hoc tests can be performed to reveal any significant differences in algorithm performance.

Nemenyi’s post–hoc procedure for multiple comparisons [43] goes further than the previous two, in that it singles out pair(s) of algorithms which point out the relevant disagreements, as well as quantifying the magnitude of these disagreements.

We have applied the Nemenyi test to obtain pairwise comparisons of the four classifiers. Any two classifiers are considered to disagree significantly if the corresponding average ranks differ by at least the Critical Difference (*CDf*) defined in Eq 10, where critical values *q*_*α*_ are based on the Studentized range statistic divided by $\sqrt{2}$. As before, *k* is the number of algorithms and *N*_*ds*_ is the number of data sets.

$$\begin{array}{c} CDf=q_{\alpha }\frac{k(k+1)}{6N_{ds}} \end{array}$$ (10)

**Fig 11:**
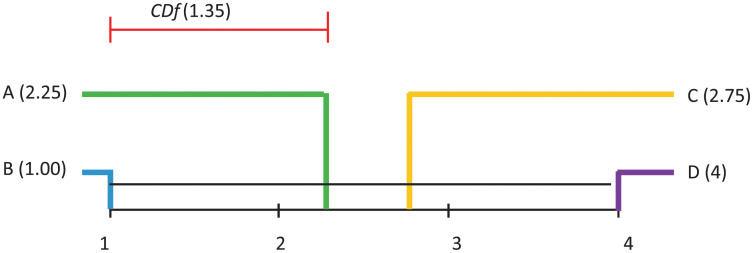
Visualisation of post hoc Nemenyi test results for all considered classification techniques over all datasets. The Avg AUC performance measure is considered. The mean ranks are depicted on the main line, while *CDf* represents the critical difference. In the case of no significant difference, the methods are furthermore connected.

**Fig 12:**
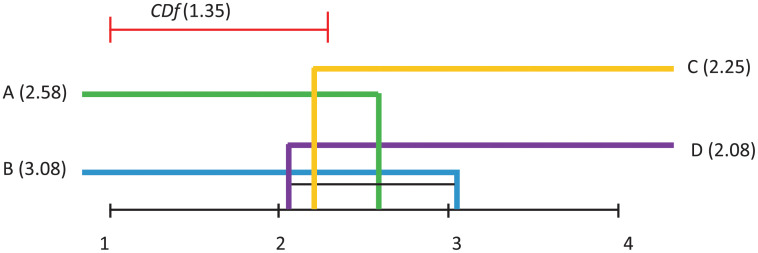
Visualisation of post hoc Nemenyi test results for all considered classification techniques over all datasets. The Avg Acc performance measure is considered. The mean ranks are depicted on the main line, while *CDf* represents the critical difference. In the case of no significant difference, the methods are furthermore connected.

**Fig 13:**
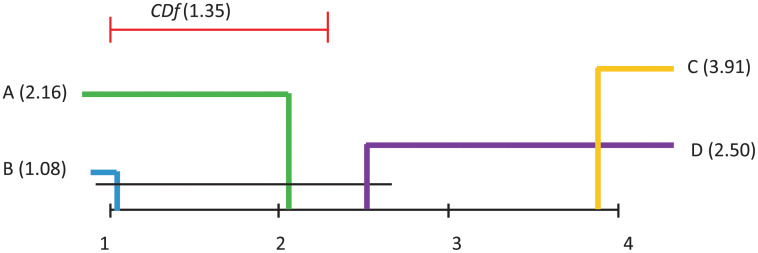
Visualisation of post hoc Nemenyi test results for all considered classification techniques over all datasets. The AUC Pareto optimal front performance measure is considered. The mean ranks are depicted on the main line, while *CDf* represents the critical difference. In the case of no significant difference, the methods are furthermore connected.

**Fig 14:**
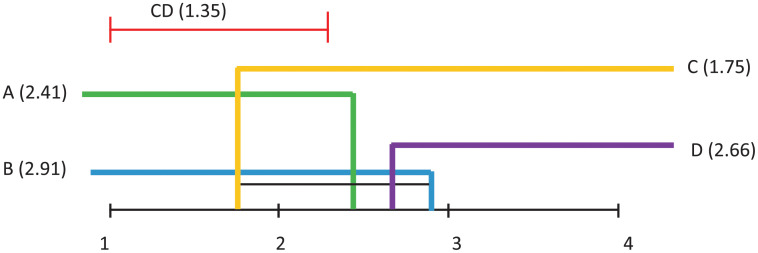
Visualisation of post hoc Nemenyi test results for all considered classification techniques over all datasets. The Avg Acc Pareto optimal front performance measure is considered. The mean ranks are depicted on the main line, while *CDf* represents the critical difference. In the case of no significant difference, the methods are furthermore connected.

Demsar [37] proposed a way of visualising the results of a post–hoc analysis, where several algorithms are compared. *CDf* diagrams (such as the ones in Figs 11–14 illustrate the ranking of each algorithm in terms of its average rank, the magnitude of the disagreement between them, and the interpretation of these rankings.

Differences in algorithm performance is considered statistically significant if algorithms are placed further apart than the specified *CDf*. The black lines in these diagrams connect groups of algorithms whose performance is not significantly different.

Subsequent to the Friedman test rejections, the Nemenyi post–hoc test compares all classifiers pairwise, and concludes that two classifiers can be considered significantly different from the performance point of view if their average ranks are different at least from the *CDf* perspective.

Fig 11, where Average AUC is considered as performance measure, indicates that some significant differences appear between: A and C, B and C, B and D.

Fig 12, where Average Accuracy is considered as performance measure, indicates that no significant differences appear between any pairs of models.

Fig 13, where the AUC of the Pareto optimal front is considered as performance measure, indicates that some significant differences appear between: A and C, B and C, B and D, C and D.

Finally, Fig 14, where AverageAccuracy is considered as performance measure, indicates that no significant differences appear between any pairs of models.

## Related work

In recent times the research for automated breast cancer recognition in digital mammography was dominated by deep learning and convolutional neural networks. The capabilities of deep learning were initially compared to those of traditional CAD methods, which rapidly revealed the promise of this new technology. The recognition results obtained by deep learning methods have been compared against those obtained by experts radiologists, indicating a good performance [3, 44, 45]. However, new improvements are possible.

Evolutionary computation represents an alternative to deep learning. Deep learning is focused on modeling what we know, whereas evolutionary computation is focused on developing new solutions. Exuberant, but directed, exploration is enabled thanks to evolutionary computation, which allows for the discovery of novel designs and behaviors. In some ways, it is the next step beyond deep learning: an intelligent system that can think outside the box. Deep learning has demonstrated its worth in automating well-known and well-described actions and abilities, but it lacks the ability to go beyond them. This is why evolutionary computation is so important for classification problems too.

In addition, because the internal learning processes as well as the resulting models are not totally visible, deep learning looks to be a “black box” due to the increasing complexity of underlying models and algorithms. Systems based on artificial intelligence must provide reliable support for society through robustness and reliability as well as compliance with legal regulations and ethical principles. Trustworthy AI brings important challenges to the development of transparent, accountable and human-centered systems, accountability-oriented technologies in terms of data collection and algorithm design [46]. In this context, the Evolutionary Computationhas the potential to balance the performance and the explainability of an intelligent system.

Recently, the use of evolutionary algorithms for training classifiers has been investigated. Genetic programming (GP) is a versatile and powerful evolutionary technique with specific characteristics that make it ideal for classifier evolution. In [47] the authors have investigated the capability of the GP approach for producing hierarchical, rule-based, classification trees. In [48] GP is used to evolve decision trees for data classification, search spaces tend to become extremely large by using data sets from the UCI machine learning data set repository. The paper [49] focuses on adapting the fitness function in GP to evolve classifiers with good individual class accuracy. In all these approaches and also many others (e.g. [50]) the evaluation of classification methods are performed by using data from UCI repository that means the image descriptors have not been taken into account, UCI providing directly the extracted features from different, medical or not, image databases. In our study, the raw images are considered and the visual descriptors are computed directly, in order to obtain a more realistic framework that can truly help a medical practitioner.

Due to the fact that classification is by definition a task consisting of multiple objectives that are usually conflicting, the use of multi-objective optimization for solving classification problems has been intensively studied. Some of the most popular algorithms are Pareto Envelope-based Selection Algorithm (PESA) [51], Strength Pareto Evolutionary Algorithm (SPEA2) [52] and Non-dominated Sorting Genetic Algorithm (NSGA-II) [24].

In [53] the authors strive to minimize both the number of mistakes and the number of tree nodes while evaluating decision trees, which is one of the early approaches to utilize GP and Pareto-based multi-objective optimization for image classification. In [54] the authors present another evolutionary multi-objective strategy for creating decision trees for picture categorization. The tree size and the training error are the two objectives to be minimized. In [26], the NSGA-II algorithm is used to suggest two novel GP-based approaches. The first method aims to reduce the GP-evolved program’s classification error and size, whereas the second one divides the single goal of reducing the classification error rate into numerous goals of lowering the error for each class. In a multi-objective GP approach to classification, two opposing goals are considered: false negative rate vs. false positive rate [55]. In the case of uneven training data, multi-objective GP has recently been employed for classification [27].

The most important shortcommings of the described approach is represented by the scarcity of assessment methodologies used for validating an MOEA approach. Such assessment is difficult because MOEAs produce more solutions (instead of a single one) by stochastic methods (that require to be validated by statistical tools). In addition, the final client could be interested in measuring in different ways the produced results. The previous described approach didn’t advance such challenges. In our numerical experiments, we searched an answer for these issues.

## Conclusions

We have seen how various statistical tests rank one or the other algorithm as better than the others in our set, for our statistical confidence level to the conventional value of 95%, and hence a corresponding significance level *α* = 0.05. Had we chosen a smaller *α* we might have obtained slightly different rankings, but it is still unlikely that we would have achieved agreement amongst the rankings.

These results are hardly surprising. They agree with Demšar’s [37] conclusion that there is no actual gold standard for comparing classes of learning or evolutionary algorithms in a single fashion. At best, one can apply the classifiers to a single data set, in which case the performance is biased by the variance, or lack thereof, within that data. When working with generic or unrelated data, however, it is hard to infer that one meta–classifier is better than any other.

In line with Wolpert’s *No Free Lunch* theorems [56], our findings highlight shortcomings in the medical image classification literature: it is inadvisable and insufficient to construct meta–classifiers and measure them using a single, favourable metric, because the effect is to skew the results.

The way to mitigate these shortcomings is to ensure an adequate choice of objectives and metrics pairs. For instance, a focus on maximising the number of true positives is directly linked with using accuracy–related objectives. Conversely, if accuracy is chosen as the generic metric, its effect is to focus the primary classification objective on maximising the positively classified data points.

Future work includes detection of cancer in digital breast tomosynthesis also and to extend the multi-objective evolutionary approach to other tasks (lession segmentation, lession stratification). Furthermore, depending on the usage scenario, a variety of medico-legal and ethical issues would need to be addressed and explained after the technical/clinical performance assessment is completed. Once all of these obstacles have been overcome, Evolutionary Computation may be expected to revolutionize the way breast cancer screening is done.

## Supporting information

S1 Text (TXT)
